# Supplementary material for: Structure of the Neisseria meningitidis Type IV pilus
Source: Nat Commun. 2016 Oct 4;7:13015. doi: 10.1038/ncomms13015 (PMC5059446; doi:10.1038/ncomms13015)
Supplement: Supplementary Information — Supplementary Figures 1 – 3 [file ncomms13015-s1.pdf]

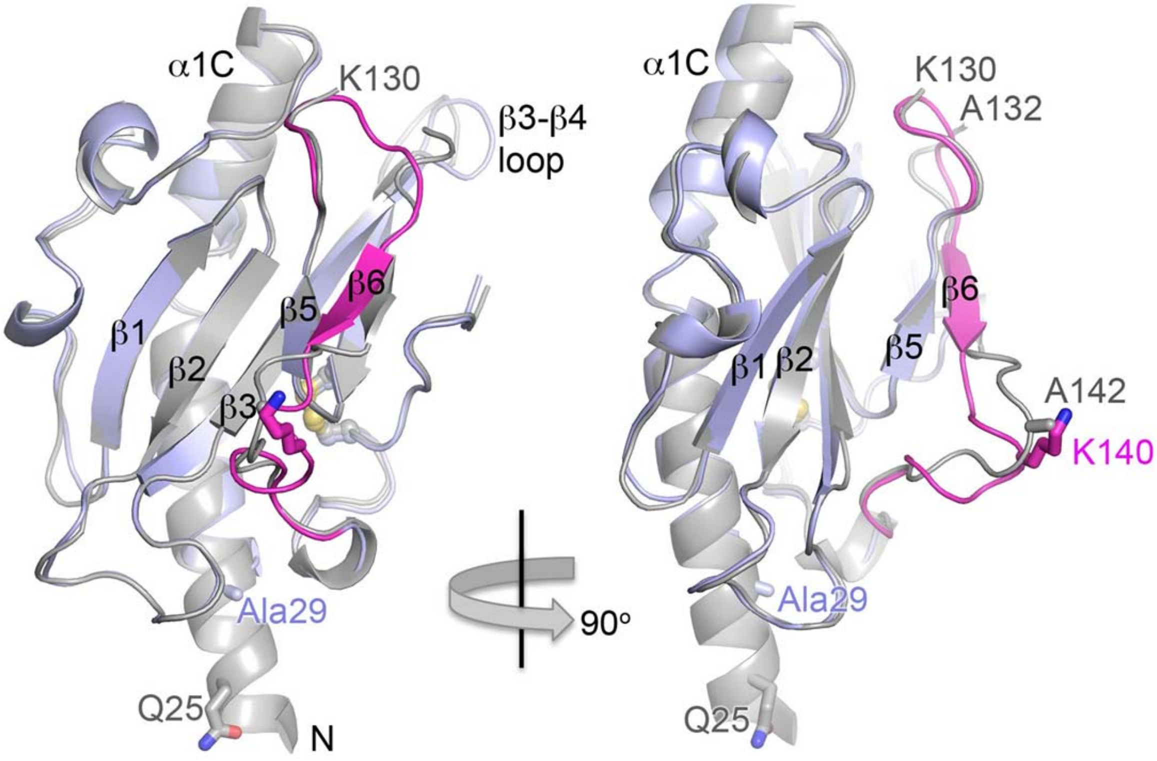

Supplementary Figure 1. Superposition of Nm PilE structures from strains 8013 and MC58. The strain MC58 PilE (4V1J, grey) is superimposed on the strain 8013 PilE structure described here (5JW8, light blue with magenta hypervariable region). The N-terminal residues corresponding to the beginning of the PilE sequences for these N-terminally truncated pilins are shown in stick representation. The disulfide bonded cysteine side chains are shown in ball-and-stick representation (sulfur colored yellow). Ala131 is not resolved in the MC58 PilE structure.

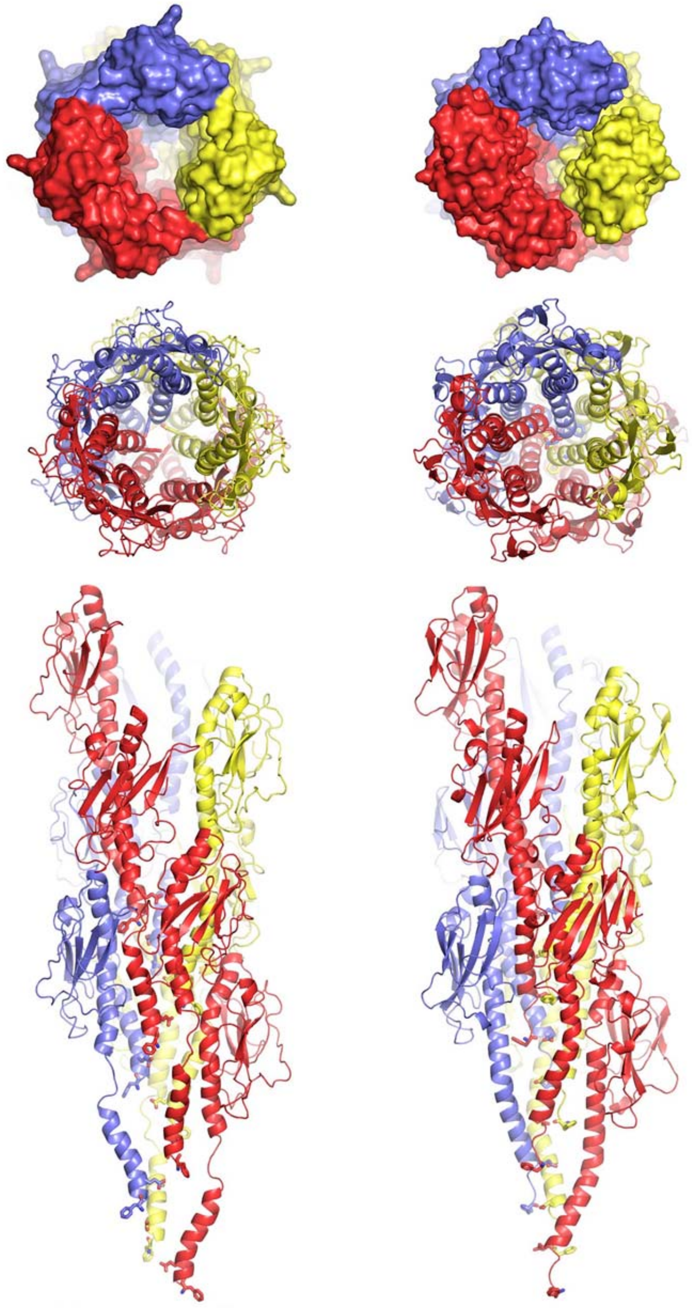

Supplementary Figure 2. Comparison of models from the ~ 6 Å Nm and 12.5 Å Ng Type IV pilus reconstructions. Both filament models are comparable in diameter (~ 6 nm) and helical symmetry: Nm T4p rise, 10.3 Å, rotation, 100.5 ^{o}; Ng T4P rise, 10.5 Å, rotation, 100.8 ^{o}. The models differ significantly in the structure and position of α1N. Subunits are colored red, yellow and blue along their 3-start helices.

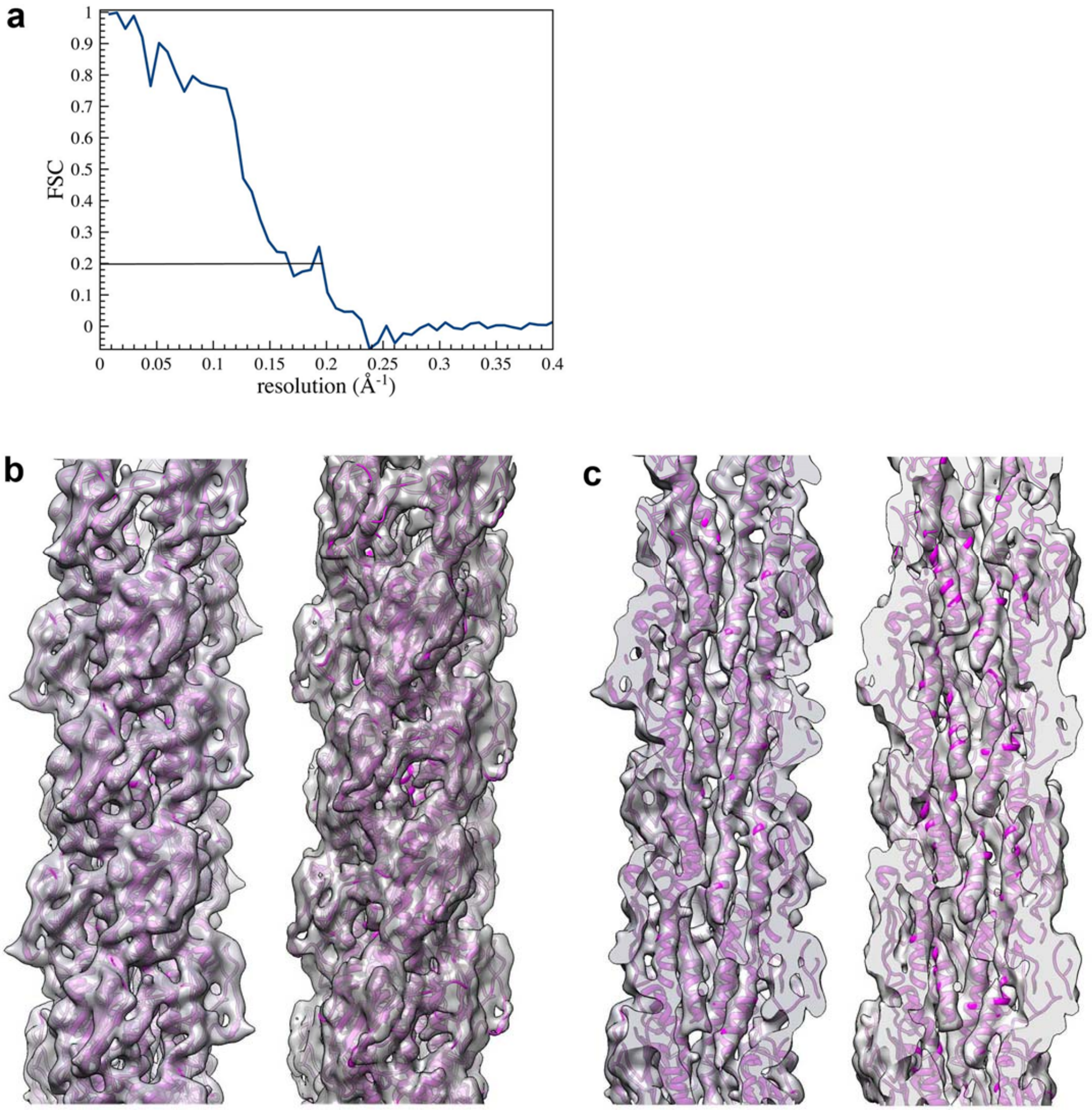

Supplementary Figure 3. Fourier shell correlation between cryoEM map and the Nm Type IV pilus model. (a) The resolution of the Nm T4P reconstruction is estimated to be ~6 Å. This estimate is reasonable when one compares the model volume filtered to 6 Å resolution with the actual reconstruction. The filtered model is shown on the left and the cryoEM map is on the right, shown **(b)** from the outside of the pilus and **(c)** in cross-section.
